# Association of high-risk neuroblastoma classification based on expression profiles with differentiation and metabolism

**DOI:** 10.1371/journal.pone.0245526

**Published:** 2021-01-19

**Authors:** Shunsuke Kimura, Masahiro Sekiguchi, Kentaro Watanabe, Mitsuteru Hiwatarai, Masafumi Seki, Kenichi Yoshida, Tomoya Isobe, Yusuke Shiozawa, Hiromichi Suzuki, Noriko Hoshino, Yasuhide Hayashi, Akira Oka, Satoru Miyano, Seishi Ogawa, Junko Takita

**Affiliations:** 1 Department of Pediatrics, Graduate School of Medicine, The University of Tokyo, Tokyo, Japan; 2 Department of Pediatrics, Graduate School of Biomedical Sciences, Hiroshima University, Hiroshima, Japan; 3 Department of Pathology and Tumor Biology, Graduate School of Medicine, Kyoto University, Kyoto, Japan; 4 Institute of Physiology and Medicine, Jobu University, Gunma, Japan; 5 Human Genome Center Institute of Medical Science, The University of Tokyo, Tokyo, Japan; 6 Department of Pediatrics, Kyoto University, Kyoto, Japan; 2nd Medical Faculty Charles University Prague and Faculty Hospital Motol, CZECH REPUBLIC

## Abstract

Neuroblastoma, the most common extracranial solid malignancy among children, originates from undifferentiated neural crest cells (NCC). Despite recent intensified treatment, high-risk patients still have a high mortality rate. To explore a new therapeutic strategy, we performed an integrated genomic and transcriptomic analysis of 30 high-risk neuroblastoma cases. Based on the expression profiling of RNA sequencing, neuroblastoma was classified into Mesenchymal (MES; *n* = 5) and Noradrenergic (ADRN; *n* = 25) clusters, as previously reported in the super-enhancer landscape. The expression patterns in MES-cluster cases were similar to normal adrenal glands, with enrichment in secretion-related pathways, suggesting chromaffin cell-like features built from NCC-derived Schwann cell precursors (SCPs). In contrast, neuron-related pathways were enriched in the ADRN-cluster, indicating sympathoblast features reported to originate from NCC but not via SCPs. Thus, MES- and ADRN-clusters were assumed to be corresponding to differentiation pathways through SCP and sympathoblast, respectively. ADRN-cluster cases were further classified into MYCN- and ATRX-clusters, characterized by genetic alterations, *MYCN* amplifications and *ATRX* alterations, respectively. MYCN-cluster cases showed high expression of *ALDH18A1*, encoding P5CS related to proline production. As reported in other cancers, this might cause reprogramming of proline metabolism leading to tumor specific proline vulnerability candidate for a target therapy of metabolic pathway. In ATRX-cluster, *SLC18A2* (VMAT2), an enzyme known to prevent cell toxicity due to the oxidation of dopamine, was highly expressed and VMAT2 inhibitor (GZ-793A) represented significant attenuation of cell growth in NB-69 cell line (high *SLC18A2* expression, no *MYCN* amplification) but not in IMR-32 cell line (*MYCN* amplification). In addition, the correlation of VMAT2 expression with metaiodobenzylguanidine (MIBG) avidity suggested a combination of VMAT2 inhibitor and MIBG radiation for a novel potential therapeutic strategy in ATRX-cluster cases. Thus, targeting the characteristics of unique neuroblastomas may prospectively improve prognosis.

## Introduction

Neuroblastoma, the most common extracranial solid malignancy among children under 15 years of age, accounts for 8%–10% of pediatric tumors [1, 2]. In general, neuroblastoma is thought to originate from undifferentiated neural crest cells (NCC), which can become any of several different cell types, depending on the location within the embryo [3]. NCCs may partially differentiate into neuroblastoma or ganglioneuroblastoma, which are malignant, or may differentiate into benign ganglioneuroma [4]. The primary site of neuroblastoma is typically the adrenal medulla or tissues that originate from the sympathetic nervous system [2]. Although low- and intermediate-risk patients generally have a favorable outcome, high-risk patients show a high mortality rate and fewer than 50% of patients have long-term survival despite recent intensified treatment [5, 6].

Several genome-wide analysis studies have been reported which aimed to improve prognosis and develop a new therapeutic strategy for high-risk neuroblastoma [2, 7, 8]. Segmental chromosomal aberration is common in high-risk neuroblastoma, such as amplification of the *MYCN* oncogene [9] deletions of chromosomes 1p, 3p, 4p, and 11q, and gains of chromosomes 1q, 2p, and 17q [10–12]. These chromosomal aberrations greatly influence the clinical course [13]. In contrast, recent high-throughput genome-wide studies have revealed that there were few recurrent somatic alterations in high-risk neuroblastoma, except *MYCN* [9] *ALK* [14–17] *ATRX* [18, 19] and *TERT* [20]. These genetic alterations do not account for the entire genetic mechanism leading to high-risk neuroblastoma. Thus, effective targeted therapies for intractable neuroblastoma remain limited.

Based on the super-enhancer landscape of neuroblastoma cell lines, there are two neuroblastoma subtypes: Noradrenergic (ADRN)-type and Mesenchymal (MES)-type, showing distinct expression patterns in core regulatory circuitry (CRC)-related genes [21, 22]. However, expression profiling of high-risk neuroblastoma is not yet fully understood. In the present study, we classified 30 cases of International Neuroblastoma Staging System (INSS) [23] Stage 4 neuroblastomas, based on expression profiles of whole transcriptome sequencing (WTS). Then we conducted a combined analysis with data on mutations and copy number alteration (CNA) to explore a new therapeutic strategy for high-risk neuroblastoma.

## Materials and methods

### Patients and materials

This study enrolled 30 specimens collected from pediatric patients (2–140 months) who had been diagnosed with neuroblastoma with INSS Stage 4 neuroblastoma and admitted to Tokyo University Hospital and various hospitals in Japan between January 2003 and December 2015 (S1 Table). Enrolled patients were treated under the various protocols. All patients and/or their parents provided written informed consent, and the Human Genome, Gene Analysis Research Ethics Committee of the University of Tokyo and other participating institutes approved the study protocols. Studies were conducted at Department of Pediatrics, The University of Tokyo, in accordance with the principles of the Declaration of Helsinki. Biopsy samples at the primary tumor site were collected from neuroblastoma patients. No matched normal samples were available. Genomic DNA and RNA of each sample was isolated from frozen biopsy samples by NucleoSpin DNA RapidLyse kit and NucleoSpin RNA kit (Macherey-Nagel Gmbh & Co., Düren, Germany) according to the manufacturer’s protocol.

### Targeted capture sequencing (TCS)

Targeted capture was performed using a SureSelect custom kit (Agilent Technologies, Palo Alto, CA, USA) as previously described [24–26] The custom bait library (U-Tokyo Onco-panel ver.1) related to pediatric cancers (S2 Table) [24] was designed to include the following: (i) all coding exons of 367 genes; (ii) untranslated regions and introns of *CD274*, *CTNNB1*, *ERG*, *ETV1*, *ETV4*, *EWSR1*, *FEV*, *FLI1*, *FOXO1*, *FUS*, *INO80D*, *NCOA1*, *NCOA2*, *NOTCH1*, *PAX3*, and *PAX7* for detecting breakpoints of structural variations; (iii) promoter and enhancer regions of *FGFR3*, *MYC* and *TERT*; (iv) microRNA genes *MIR100*, *MIRLET7A1*, *MIRLET7A2*, *MIRLET7A3*, *MIRLET7B*, *MIRLET7C*, *MIRLET7D*, *MIRLET7E*, *MIRLET7F1*, *MIRLET7F2*, and *MIRLET7G*; and (v) 3,527 positions of single nucleotide polymorphisms (SNP) for copy number analysis to generate genome-wide allele-specific copy number profiles. We selected 381 targeted genes and regions, not including the SNP positions, to include the following: (a) genes adopted in more than one of the following existing gene panels: MSK-IMPACT27 CMS400 (Life Technologies, Carlsbad, CA, USA), FoundationOne (Foundation Medicine, Cambridge, MA, USA), or the Human Comprehensive Cancer Panel (QIAGEN, Hilden, Germany); (b) the most frequently mutated 20 genes in each type of malignancy according to the Catalogue of Somatic Mutations in Cancer (COSMIC) v78; and (c) genes that were recurrently affected in pediatric malignancies, including neuroblastoma, hepatoblastoma, pleuropulmonary blastoma, rhabdomyosarcoma, Ewing’s sarcoma, and germ cell tumor.

Captured targets were subjected to sequencing using a HiSeq 2000 or 2500 platform (Illumina, San Diego, CA, USA) with a standard 125-bp paired-end read protocol according to the manufacturer’s instructions. With mean depths of 411 reads (range = 267–575, S3 Table), sequence alignment and mutation calling were performed using our in-house pipeline “Genomon v.2.5.0,” as previously described [25] Reads with a mapping quality score of <25, a base quality score of <30, or five or more mismatched bases were excluded from the analysis. Candidate mutations with a variant allele frequency in tumor samples ≥0.1 and an EBcall [27] (Empirical Bayesian mutation calling) *P* ≤ 1 ×10^−3^ in coding regions were adopted, and filtered by excluding the following: (i) synonymous mutations and variants without complete ORF information; (ii) known variants listed in the 1000 Genomes Project (Oct 2014 release); NCBI SNP database (dbSNP) build 131, National Heart, Lung, and Blood Institute (NHLBI) Exome Sequencing Project (ESP) 6500, the Human Genome Variation Database (HGVD; October 2016 release), or our in-house SNP database; (iii) variants present only in unidirectional reads; (iv) variants occurring in repetitive genomic regions; (v) variants with <5 supporting reads in tumor samples; and (vi) all variants found in non-paired normal samples (*n* = 30) showing an allele frequency of >0.0025. Finally, mapping errors were removed by visual inspection on the integrative genomics viewer browser [28] Copy numbers were calculated by allele frequencies and sequence depths of SNPs using our in-house pipeline “CNACS” [29, 30] We defined amplification, gain, or loss of genes and segments when calculated signal values were >10, >2.75 or <1.25 (<0.75 on the X chromosome of male subjects), respectively. Significant CNAs were identified using GISTIC 2.0 (*q* < 0.1).

### Whole transcriptome sequencing (WTS)

Total RNA was assessed for integrity and concentration using an Agilent 4200 TapeStation system. All samples had an RNA integrity number higher than 6.5. Libraries for RNA-seq were prepared using the NEBNext Ultra RNA Library Prep kit for Illumina (New England BioLabs, Beverly, MA, USA). Normalized count data, obtained by Genomon v.2.5.0 and the R software package DESeq2, were subjected to cluster analysis. We ascertained cluster stability via consensus clustering by using the top 1,000 most variable genes on the basis of median absolute deviation, with 1,000 iterations, using the R package ConsensusClusterPlus. The R software package Rtsne was used to map the samples to tSNE plot. Heatmaps were generated by the R package pheatmap using count data. Differentially expressed genes were extracted using the R package DESeq2, and pathway analysis was performed using Metascape (http://metascape.org).

### Data

The raw data of sequencing are available from the DNA Data Bank of Japan (DDBJ) (accession number hum0035, JGAS00000000246). The results published here are in part based upon data generated by the Therapeutically Applicable Research to Generate Effective Treatments (https://ocg.cancer.gov/programs/target) initiative, phs000467. The data used for this analysis are available at https://portal.gdc.cancer.gov/projects (TARGET-NBL).

### Cell proliferation assay

NB-69 and IMR-32 cell lines were obtained from Dr. Yasuhide Hayashi (Jobu University). Mycoplasma infection and short tandem repeat were not tested. To select representative cell lines for each cluster (MYCN- and ATRX-cluster), we used public data of transcriptomic profiling in 39 neuroblastoma cell lines (GSE89413) [31]. Cells were seeded in triplicate at the concentrations of 3,000 cells/well in 96-wells plate. After 24 hours, cells were treated with DMSO (control), Tolcapone (COMT inhibitor, Sigma #SML0150) or GZ-793A (VMAT2 inhibitor, Sigma #SML0851). Cell proliferation was measured by using Cell Counting Kit-8 (Sigma, #96992) at 24, 48, 72, 96 hours after treatment. Cell proliferation rate was calculated by comparing the absorbance before and after the treatments.

### Statistical analysis

Statistical analyses were performed using R v3.4.0 software. Adjusted *P* values of differentially expressed genes were calculated by DESeq2 and were considered statistically significant at adj*P* < 0.01. *P* values and q values of pathway analysis were analyzed by using Metascape and were considered statistically significant at q < 0.01. Statistical significance of gene expression level and variant allele frequency were assessed using the Wilcoxon rank-sum test. The resulting values were considered statistically significant at *P* < 0.05.

## Results

### Mutation and copy number analysis

**Fig 1:**
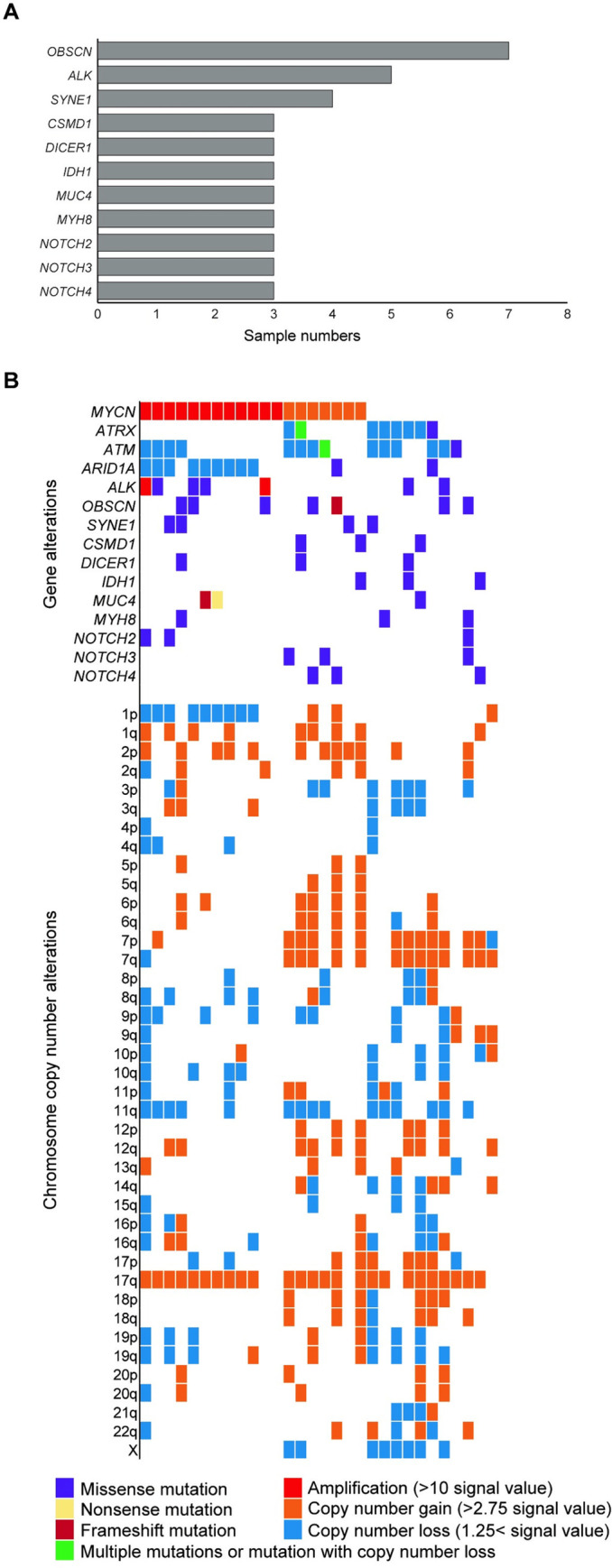
TCS in 30 cases of INSS Stage 4 neuroblastoma. (A) Recurrently detected gene alterations (> 10%) by TCS for 381 pediatric cancer-related genes. Since only tumor samples were available, candidate variants were filtered according to our previous study to estimate somatic mutations. (B) Mutational landscape in 30 cases of neuroblastoma, along with copy number alterations based on TCS data. TCS, targeted capture sequencing. Broad and focal deletions and amplifications in the coding regions were also annotated in “Gene alteration”.

We performed TCS for 381 pediatric cancer-related genes and regions (U-Tokyo Onco-panel ver.1) [24] (S2 Table) on 30 cases of INSS Stage 4 neuroblastoma (S1 Table). Each neuroblastoma sample harbored a mean of 4.2 variants (range = 1–10) (S4 Table). Variants in *OBSCN* (23.3%) were the most frequent, followed by *ALK* variants (16.7%) (Fig 1A and S1 Fig). Broad gains of chromosomes 17q (83.3%), 1q (40%), and 2p (40%), and deletions of chromosomes 11q (50%) were commonly detected (Fig 1B). Twelve cases (40%) possessed focal amplification of the *MYCN* oncogene. Alterations of *ATM* (46.7%), *ARID1A* (36.7%), and *ATRX* (26.7%), including broad and focal deletions in the coding region (annotated in blue) and mutations (annotated in green and purple), were also observed as previously reported [7, 12, 32]. Alteration in *TERT* gene region was not detected. Consistent with previously reported [33], RAS and p53 pathways were frequently affected (S1 Table). The amplification of the *MYCN* oncogene and alterations in *ATRX* appeared to be mutually exclusive (Fig 1B).

### Unsupervised consensus clustering based on expression profiles

**Fig 2:**
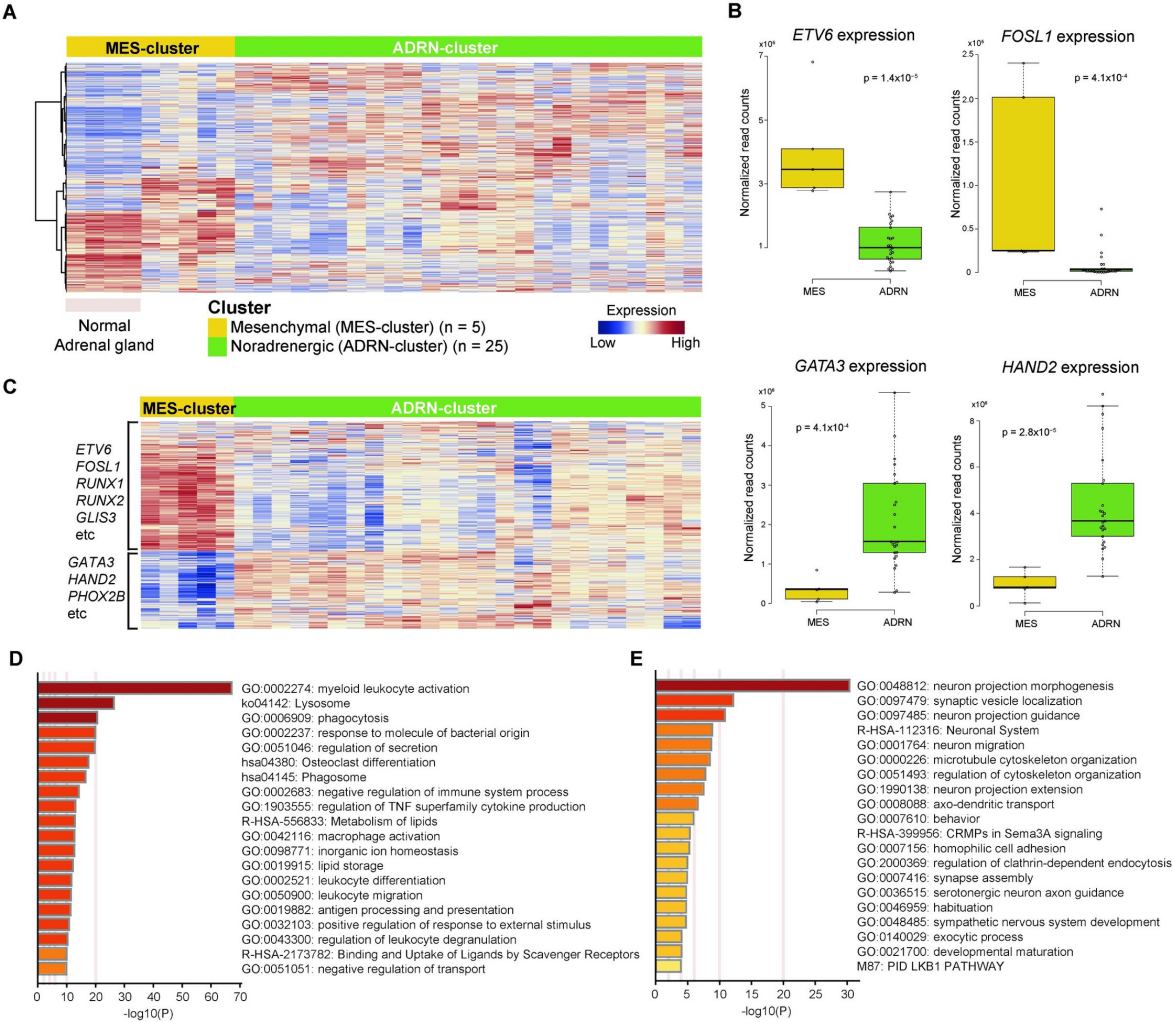
Classification of 30 cases of Stage 4 neuroblastoma based on expression profiles. (A) Unsupervised consensus clustering of total 34 samples (30 neuroblastoma and four normal adrenal glands; GSE93499, GSE88682, GSE88668 [34]) identified two distinct subgroups, MES- and ADRN-clusters. Five cases of MES-cluster neuroblastoma showed similar expression profiles to normal adrenal gland samples. (B) The expression of core genes indicated two subtypes. Normalized expression was calculated from read counts on each gene with DESeq2 software. The *P*-value was calculated using the Wilcoxon rank-sum test. The mean and 25th and 75th percentiles are represented in the box plots by the midline and box edges, respectively, and whiskers extend to 1.5 times the interquartile range. (C) Supervised hierarchical clustering of 30 neuroblastoma cases using the same gene set used for classification of MES- and ADRN-clusters in previous reports [21, 22]. Pathway analysis with Metascape software by using top 500 differentially expressed genes in (D) ADRN-cluster and (E) MES-cluster. MES, Mesenchymal; ADRN, Noradrenergic.

We performed gene expression profiling of 30 individuals with INSS Stage 4 neuroblastoma, in addition to publicly-available expression data of normal adrenal gland tissues (GSE93499, GSE88682, GSE88668), to characterize neuroblastoma in genomic terms [34] We obtained two stable clusters using unsupervised consensus clustering which validated in tSNE plot (S2 Fig). Importantly, five (17%) cases of neuroblastoma had similar expression profiles to the normal adrenal gland (Fig 2A). Among these five cases, information about the primary tumor site was available in four cases; three were in the adrenal gland, and one in the retroperitoneum (S3 Fig and S1 Table). Although samples of these five cases were obtained from a biopsy of the primary site, results of CNA and variant allele frequency detected by TCS were compatible with the rest of 25 neuroblastoma cases in the other cluster (S4 Fig and S4 Table). Therefore, these five samples seemed to have sufficient numbers of tumor cells. We concluded that the effects of contaminated normal adrenal gland cells were not strong compared to the rest of the samples.

Comparison of expression profiles of these two clusters revealed that several transcription factors involved in CRC, including *ETV6* and *FOSL1*, were highly expressed in the cluster as previously described [22], showing similar expression patterns to normal adrenal glands (Fig 2B and S5A Fig). Cases in the other cluster also showed a high expression of CRC-related genes in neuroblastoma, such as *GATA3* and *HAND2* (Fig 2B and S5B Fig). We validated the expression profiles of these two clusters using differentially expressed genes between ADRN- and MES-types reported in the classification of super-enhancer-associated transcription factor networks (S5 Table) [21, 22] As a result, expression patterns of the cluster showing similar expression profiles to normal adrenal glands corresponded with the MES type, whereas the other cluster corresponded with the ADRN type (Fig 2C). These two subtypes, MES- and ADRN-clusters were validated by using expression profiles of 94 cases with high-risk neuroblastoma in the Therapeutically Applicable Research to Generate Effective Treatment (TARGET) database (S6 Fig and S6 Table). Thus, based on expression profiling, INSS Stage 4 neuroblastoma in the present study was classified into two subtypes, MES- and ADRN-clusters, and cases in the MES-cluster presented similar expression patterns to normal adrenal glands.

### Pathway analysis in MES- and ADRN-clusters

For further characterization of MES- and ADRN-clusters, we performed pathway analysis using the 500 top-ranked differentially expressed genes between these clusters (S7 Table). In the MES-cluster, pathways related to secretion and vesicles were enriched significantly, whereas the ADRN-clusters had enriched neuron- and synapse-related pathways (Fig 2D and 2E). These results indicated that the individual cases in MES- and ADRN-clusters harbored features of chromaffin cells and the sympathetic nervous system, respectively. About 80% of chromaffin cells, which are the main components of the adrenal medulla, were reported to be generated from NCC-derived Schwann cell precursors (SCPs) later in embryonic development [35] Furthermore, SCPs and sympathoblasts originate from unique single NCCs traced from E8.5 [35] Thus, the MES- and ADRN-clusters appeared to correspond to differentiation pathways through SCP and sympathoblast cells, respectively, in the development model. These differences might be involved in expression patterns and the development of neuroblastoma of each cluster (Table 1). In fact, all cases that originated from the neck and mediastinum, rather than the adrenal gland or the retroperitoneum, were classified into the ADRN-cluster, while all MES-cluster cases originated from the adrenal gland or the retroperitoneum (S1 Table).

**Table 1:** Characterization of ADRN- and MES-clusters considering of differentiation.

	Noradrenergic (ADRN)	Mesenchymal (MES)
Time of differentiation	Early	Late
Origin	Neural crest cell (NCC)	Schwann cell precursor (SCP)
Cell	Sympathetic neuron	Chromaffin cell
Tissue	Sympathetic nervous system	Adrenal gland
Synapse	Yes	No

### Further classification of ADRN cluster based on expression profiles

**Fig 3:**
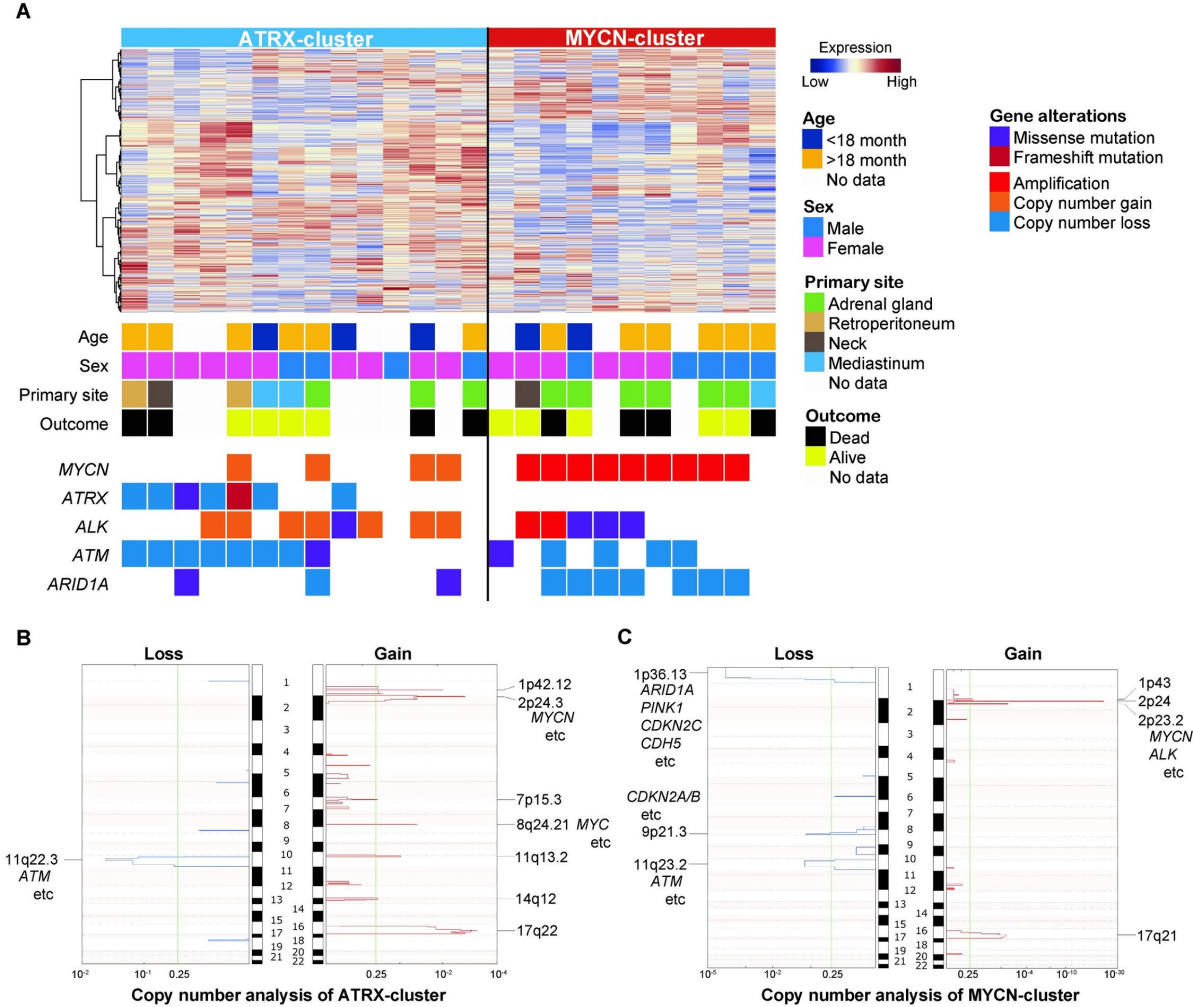
Further classification of 25 neuroblastoma cases in ADRN-cluster. (A) Unsupervised consensus clustering classified 25 cases of ADRN-cluster neuroblastoma into two subgroups, ATRX- and MYCN-clusters, characterized by genetic alterations. Clinical data and mutational landscapes are also shown. The results of copy number analysis based on TCS data in (B) ATRX-cluster and (C) MYCN-cluster are shown. TCS, targeted capture sequencing. Broad and focal deletions/gains and amplifications in the coding region were also annotated.

We performed unsupervised consensus clustering again only for the ADRN-cluster neuroblastoma cases (n = 25) to further characterize this cluster that accounted for 80% of INSS Stage 4 neuroblastoma cases in the present study. With the top 1000 most variable genes, the ADRN-cluster was divided into two groups by 2^nd^-unsupervised consensus clustering and tSNE plot (S7 Fig). Based on genetic alterations detected by TCS, these two subtypes were characterized into MYCN- and ATRX-clusters (Fig 3A). The MYCN-cluster contained all the cases with *MYCN* amplification, as well as frequent alterations of *ALK* and *ARID1A* including focal and broad aberrations in the coding region. The ATRX-cluster contained all of the cases with *ATRX* abnormalities and frequent *ATM* alterations involving coding region, although no cases with *MYCN* focal amplification were classified into this cluster. CNA revealed frequent segmental chromosomal aberration in the ATRX-cluster, especially deletions of chromosome 11q, including the *ATM* locus, and gains of chromosomes 2p and 17q (Fig 3B and S7 Table). In contrast, MYCN-cluster featured deletions of chromosome 1p with a gain of chromosome 2p including the *MYCN* and *ALK* loci (Fig 3C and S7 Table). Thus, neuroblastoma cases in the ADRN-cluster were classified into two genetically distinct clusters, MYCN- and ATRX-clusters.

### Differentially expressed genes between MYCN- and ATRX-clusters

**Fig 4:**
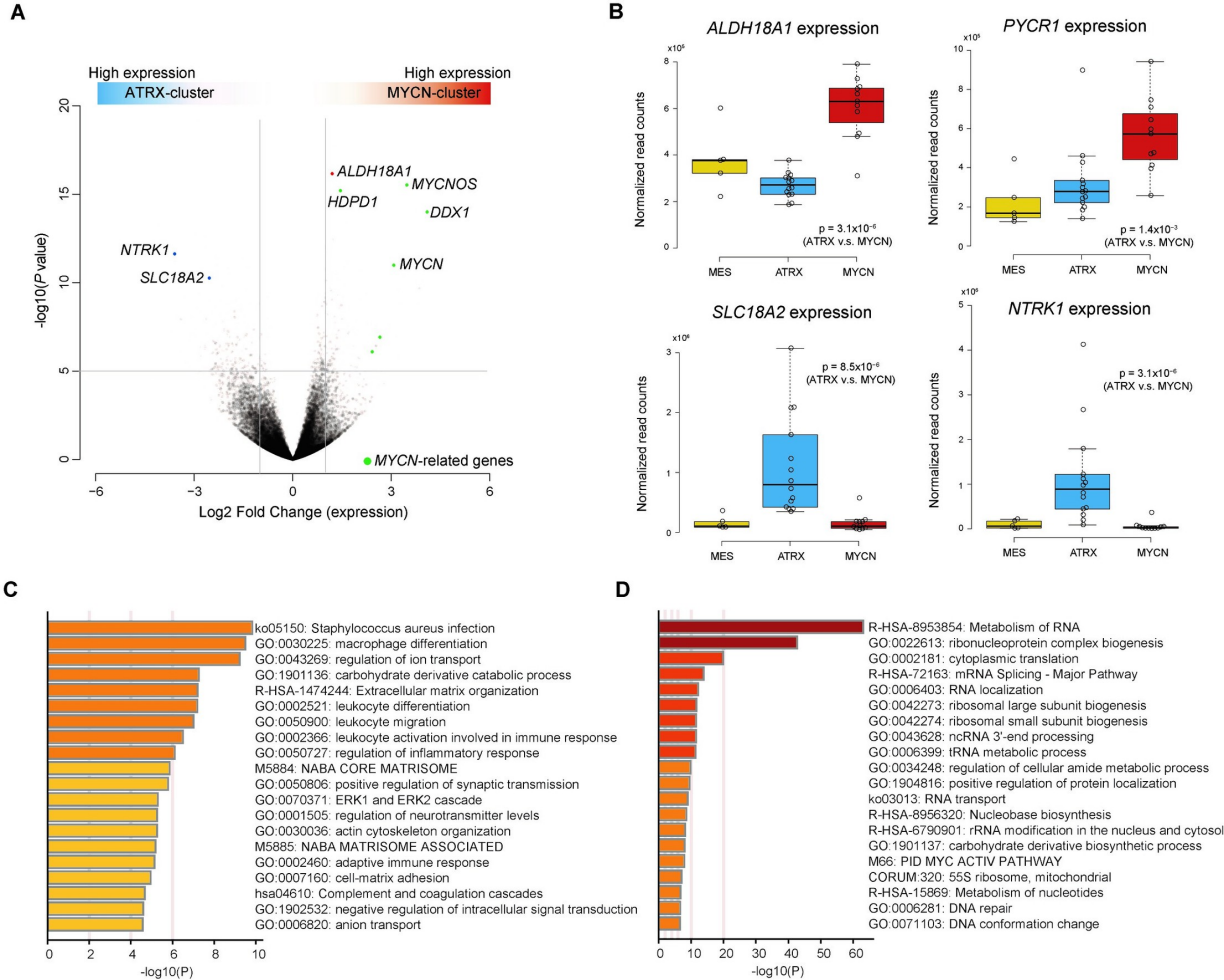
Characterization of gene expression between MYCN- and ATRX-clusters. (A) Differentially expressed genes between MYCN- and ATRX-clusters based on normalized WTS read counts. Green dots represent genes regulated by MYCN. (B) Expression of genes in MES-, ATRX- and MYCN-clusters. Normalized expression was calculated from read counts on each gene with DESeq2 software. The *P*-value between ATRX- and MYCN-clusters was calculated using the Wilcoxon rank-sum test. The mean and 25th and 75th percentiles are represented in the box plots by the midline and box edges, respectively, and whiskers extend to 1.5 times the interquartile range. Pathway analysis with Metascape software by using top 500 differentially expressed genes in (C) ATRX-cluster and (D) MYCN-cluster. MES, Mesenchymal.

Pathway analysis using the 500 top-ranked differentially expressed genes between the MYCN- and ATRX-clusters (S8 Table) revealed significant enrichment of ribosome-related pathways in the MYCN-cluster (Fig 4). This result was consistent with the fact that MYC family proteins were regulators of ribosome biogenesis and translation [36] In addition to *MYCN* and ribosome-related pathway genes, *ALDH18A1* was expressed significantly in the MYCN-cluster compared to the ATRX-cluster (Fig 4A). *ALDH18A1* encodes P5CS, an enzyme involved in the conversion of glutamate to pyrroline-5-carboxylate (P5C). This enzyme is important for producing proline from glutamate, as well as PYCR1, which also highly expressed in the MYCN-cluster (Fig 4B). These results might indicate reprogramming of proline and glutamine metabolism, increasing proline biosynthesis in the MYCN-cluster.

In the ATRX-cluster, *SLC18A2* encoding the protein VMAT2 was significantly expressed (Fig 4) compared to the MYCN-cluster. VMAT2 is a vesicular monoamine transporter that accumulates cytosolic monoamines, such as dopamine, into synaptic vesicles. This action was consistent with the pathway analysis results, which enriched vacuole- and vesicle-related pathways in the ATRX-cluster (Fig 4C and 4D). Therefore, metabolic pathways related to dopamine in the ATRX-cluster might be distinct from other neuroblastoma cases.

### Targeting the dopamine related metabolic pathways

**Fig 5:**
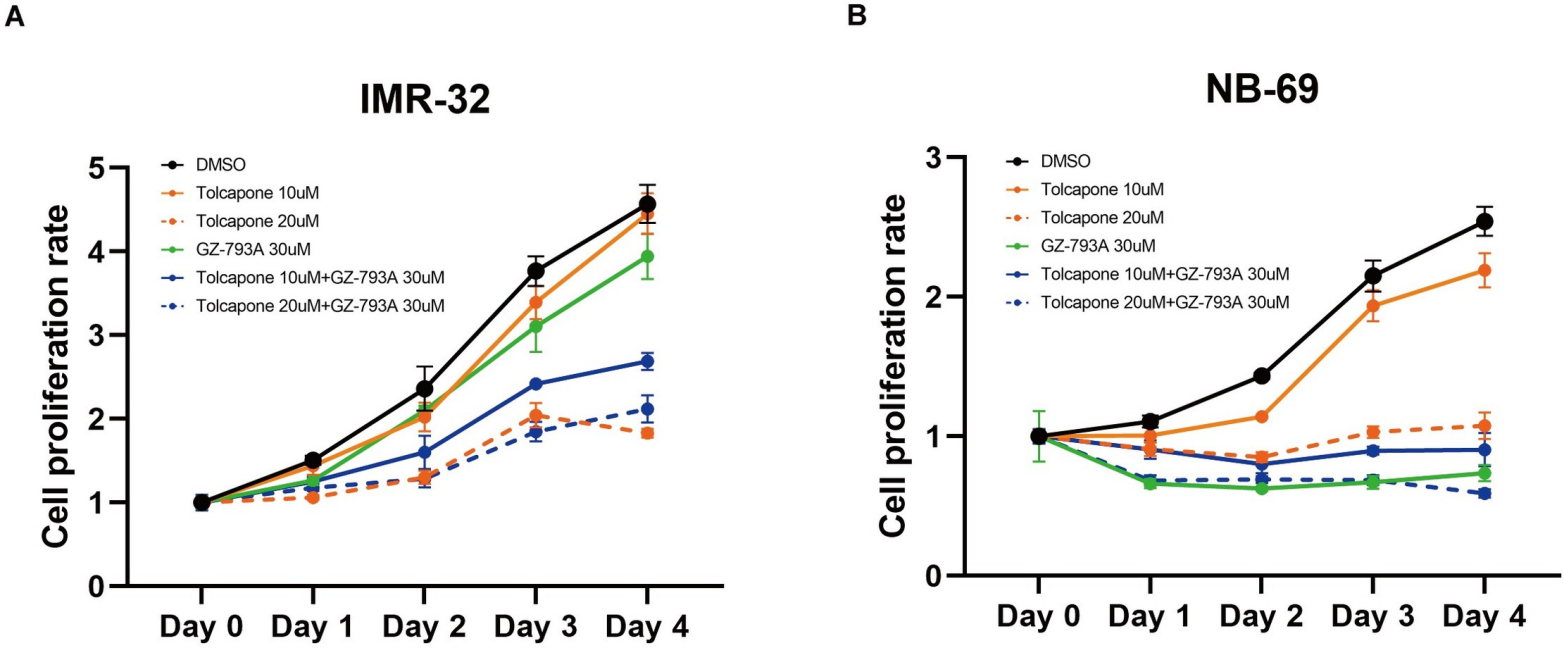
Efficacy of VMAT2 and COMT inhibitor in neuroblastoma cell lines. (A) IMR-32 (MYCN-cluster with MYCN amplification) and (B) NB-69 (ATRX-cluster with high SCL18A2 [VMAT2] expression) cells were examined in triplicate. Cells were treated with DMSO, GZ-793A (VMAT2 inhibitor, Sigma #SML0851), or Tolcapone (COMT inhibitor, Sigma #SML0150) and examined cell proliferation in each condition by using Cell Counting Kit-8 (Sigma, #96992) at 24, 48, 72, 96 hours after treatment. Mean (± SEM) cell proliferation rate was calculated by comparing detected absorbance before and after treatment.

To explore the vulnerability in the metabolic pathways related to dopamine in the ATRX-cluster, we examined the efficacy of GZ-793A (VMAT2 inhibitor) and/or Tolcapone (catechol-O-methyltransferase [COMT] inhibitor) in representative cell lines for each cluster, NB-69 (ATRX-cluster with high *SLC18A2* [VMAT2] expression without *MYCN* amplification) and IMR-32 (MYCN-cluster with *MYCN* amplification), based on the MYCN amplification status and gene expression profiles of publicly available WTS data (GSE89413) [31] because VMAT2 and COMT are enzymes to protect cells from excess reactive oxygen species (ROS) by degradation of monoamines [37, 38]. Consistent with the result from expression profiles, GZ-793A treatment significantly suppressed cell proliferations in NB-69 but not in IMR-32 (Fig 5). In contrast, Tolcapone treatment attenuated cell proliferation only in higher concentration (20μM) but not 10μM for both NB-69 and IMR-32 (Fig 5). Combination of GZ-793A and Tolcapone represented efficacy even in lower concentration of Tolcapone (10μM) in IMR-32, however, this synergistic effect was not observed in higher concentration (20μM) (Fig 5).

## Discussion

In the present study, we classified INSS Stage 4 neuroblastoma into two subtypes, MES- and ADRN-clusters, based on WTS expression profiles. These two clusters were validated with independent large cohort and consistent with the previous classification of the super-enhancer landscape of neuroblastoma cell lines [21, 22]. A small set of core transcription factors forms an interconnected auto-regulatory loop, CRC, which is often driven by super-enhancers [39]. ADRN-type neuroblastoma, showing sympathetic noradrenergic identity, was characterized by CRC modules formed by several transcription factors, such as HAND2, PHOX2B, and GATA3, leading to high expression of these genes. CRC modules, including AP-1 transcription factors, defined the MES- subtype [21, 22]. Because the formation of these CRCs is associated with differentiation, the pathway analysis results between MES- and ADRN-clusters might provide information about the origin of neuroblastomas. In the present study, enrichment of secretion- and vesicle-related pathways in the MES-cluster represented the features of chromaffin cells, composing about 80% of the adrenal medulla. In contrast, neuron- and axon-related pathways were enriched in the ADRN-cluster, which indicated sympathetic nervous system features. Furthermore, primary lesions in MES-cluster cases were limited to the adrenal gland or the retroperitoneum, whereas ADRN-cluster cases occurred in tissues originating from the sympathetic nervous system. These results were consistent with previous reports showing that most chromaffin cells originated from SCPs rather than from sympathoblasts, although both SCPs and sympathoblasts are NCC-derived [35]. Thus, there was a strong correlation between differentiation and classification of super-enhancer or expression profiles in neuroblastoma.

Primary neuroblastoma is a mixture of both MES- and ADRN-type cells, with a balance toward the ADRN type, in most samples [21, 22]. Because of intratumor heterogeneity and the usage of bulk biopsy samples, the classification of expression profiles in the present study might indicate the main cell type component in each neuroblastoma sample. Therefore, we need to be careful to say that our classification, MES- and ADRN-clusters, is directly associated with the origin of neuroblastoma. There are two possible reasons for the intratumor heterogeneity of neuroblastoma (containing both MES- and ADRN-type cells). First, critical alterations leading to neuroblastoma might occur in NCCs before differentiating into SCPs. In this case, mutated NCCs might differentiate into both MES- and ADRN-types. Second, critical alterations may occur in a later development stage, when NCC derivatives have been already determined to become either MES- or ADRN-type cells. In this case, either of the committed MES- or ADRN-type cells might interconvert to the other type. In fact, ADRN cell lines transitioned toward MES-type profiles upon chemotherapy [21, 22]. To elucidate the relevance of intratumor heterogeneity and differentiation, further analyses including single-cell analysis might be required.

Cases in the ADRN-cluster were classified into MYCN- and ATRX-clusters in the present study. MYCN-cluster cases showed high expression in *ALDH18A1* (encoding P5CS) and *PYCR1*, important enzymes in converting glutamate to proline. In Burkitt lymphoma, MYC suppressed POX/PRODH expression and increased P5CS and PYCR1, leading to reprogramming of proline and glutamine metabolism [40]. This tumor metabolic reprogramming contributes to tumor cell proliferation despite tumor-specific proline vulnerability as a compensatory mechanism [41]. In invasive breast carcinoma and kidney cancers, extensive proline production is necessary to maintain tumorigenic growth because tumor cell proliferation depends on proline [41]. As a result, high expression of *PYCR1* was induced in these cancer cells. Similar to these cancers, neuroblastoma especially in MYCN-cluster might possess a tumor-specific proline vulnerability, which could be used as a target metabolic pathway for treatment.

On the other hand, *SLC18A2* encoding VMAT2 was expressed significantly in ATRX-cluster cases and its inhibition with GZ-793A (VMAT2 inhibitor) represented significant attenuation of cell proliferation. VMAT2 is a membrane protein that transports monoamines from the cytosol into synaptic vesicles. Production of monoamines such as dopamine and noradrenaline is a hallmark of neuroblastoma. These monoamines are degraded by enzymes, COMT and monoamine oxidase (MAO), and are finally excreted as monoamine metabolites homovanillic acid and vanillylmandelic acid [42, 43] Excess free dopamine in the cytosol undergoes oxidation [38] producing ROS [37]. Since this generation of ROS induces cytotoxicity and neurodegeneration, COMT, MAO, and VMAT2 play an important role in preventing dopamine oxidation in dopaminergic neurons [38]. Therefore, Tolcapone, a potent COMT inhibitor to treat Parkinson’s disease, was reported in neuroblastoma cell lines to induce oxidative stress leading to caspase-3-mediated apoptosis and to inhibit tumor proliferation [44]. The efficacy of Tolcapone in neuroblastoma cell lines was also demonstrated in the present study and showed synergy effect with VMAT2 inhibitor in MYCN-cluster representative IMR-32 cell line. However, this synergistic effect was not observed when treated with higher Tolcapone concentration (20μM), suggesting that MYCN-cluster phenotype might use additional pathways for their proliferation and survival because Tolcapone and VMAT2 inhibitor target the same monoamine metabolism pathway. Furthermore, a report from the Children’s Oncology Group showed correlation of metaiodobenzylguanidine (MIBG) avidity with high VMAT2 expression in neuroblastoma cases without *MYCN* amplification [45]. Thus, the combination of VMAT inhibitor with COMT/MAO inhibitor (MYCN-cluster) or MIBG radiation therapy (ATRX-cluster) might be a potential therapeutic strategy for treating neuroblastoma cases.

**Fig 6:**
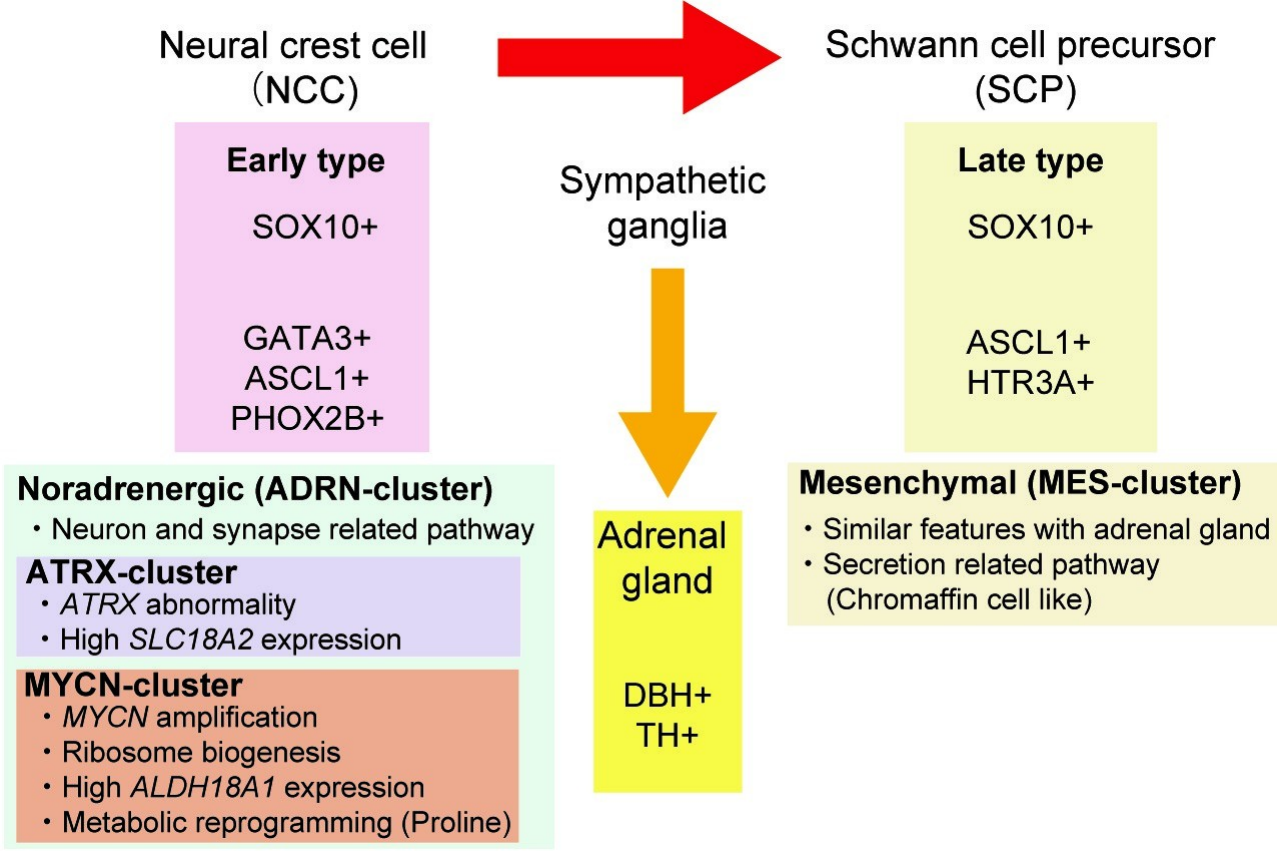
Summary of three subtypes of INSS Stage 4 neuroblastoma based on expression profiles. Neuroblastoma was firstly classified into MES- and ADRN-clusters, which suggested the difference of maturation from NCC (originate from SCPs in MES; sympathoblasts-like in ADRN). Typically expressed genes during these differentiation courses are shown in the box. The ADRN-cluster was further classified into ATRX- and MYCN-clusters, suggesting metabolic vulnerability in each cluster. MES, Mesenchymal; ADRN, Noradrenergic; SCP, Schwann cell precursors; NCC, neural crest cells; COMT, catechol-O-methyltransferase; MAO, monoamine oxidase; MIBG, metaiodobenzylguanidine.

In the present study, 30 cases with INSS Stage 4 neuroblastoma was classified into MES- and ADRN-clusters based on expression profiles. These two clusters showed association with the differentiation process and the origin of neuroblastoma (Fig 6). Further classification of the ADRN-cluster identified MYCN- and ATRX-clusters, characterized by genetic alterations and metabolism with potential therapeutic strategy. Treatment of high-risk neuroblastoma has already been intensified to the maximum limit without exceeding toxicity levels harmful to the patients. Thus, targeting metabolic reprogramming distinct in each cluster might be helpful for the development of a new therapeutic strategy for high-risk neuroblastoma.

## Supporting information

S1 Fig Sample numbers of detected mutations by targeted capture sequencing. (TIF)

S2 Fig Expression profiles of 30 NBL cases and 4 normal adrenal glands. (TIF)

S3 Fig Primary sites of neuroblastoma in each cluster. (TIF)

S4 Fig Comparison of variant allele frequency (VAF) for detected variants in ADRN- and MES-cluster. (TIF)

S5 Fig The expression of transcription factor genes participates in core regulatory circuitries in (A) MES-cluster and (B) ADRN-cluster.
(TIF)

S6 Fig Validation of MES- and ADRN-cluster in TARGET cohort. (TIF)

S7 Fig Expression profiles of 25 NBL cases of ADRN-cluster. (TIF)

S1 Table Clinical and mutational information of analyzed 30 Stage 4 neuroblastoma cases. (XLSX)

S2 Table Bait design for targeted capture sequencing. (XLSX)

S3 Table Quality control of targeted capture sequencing. (XLSX)

S4 Table Mutation calls of targeted capture sequencing. (XLSX)

S5 Table Differentially expressed gene sets reported in previous studies. (XLSX)

S6 Table Clinical and mutational information of analyzed 94 Stage 4 neuroblastoma cases in TARGET cohort. (XLSX)

S7 Table Top 500 differentially expressed genes between MES- and ADRN-clusters. (XLSX)

S8 Table Results of copy number analysis of ATRX- and MYCN-clusters using GISTIC software. (XLSX)

S9 Table Top 500 differentially expressed genes between ATRX- and MYCN-clusters. (XLSX)

S10 Table Top 500 differentially expressed genes between ATRX- and MYCN-clusters in TARGET cohort. (XLSX)

## References

[1] Tolbert VP, Coggins GE, Maris JM. Genetic susceptibility to neuroblastoma. Curr Opin Genet Dev. 2017;42: 81 90. 10.1016/j.gde.2017.03.008 28458126 PMC5604862

[2] Cheung N-KV, Dyer MA. Neuroblastoma: developmental biology, cancer genomics and immunotherapy. Nat Rev Cancer. 2013;13: 397 411. 10.1038/nrc3526 23702928 PMC4386662

[3] Mayor R, Theveneau E. The neural crest. Development. 2013;140: 2247–2251. 10.1242/dev.091751 23674598

[4] Lonergan GJ, Schwab CM, Suarez ES, Carlson CL. From the Archives of the AFIP. Radiographics. 2002;22: 911 934. 10.1148/radiographics.22.4.g02jl15911 12110723

[5] Matthay KK, Reynolds CP, Seeger RC, Shimada H, Adkins ES, Haas-Kogan D, et al Long-term results for children with high-risk neuroblastoma treated on a randomized trial of myeloablative therapy followed by 13-cis-retinoic acid: a children’s oncology group study. J Clin Oncol. 2009;27: 1007 1013. 10.1200/JCO.2007.13.8925 19171716 PMC2738615

[6] Ladenstein R, Pötschger U, Pearson A, Brock P, Luksch R, Castel V, et al Busulfan and melphalan versus carboplatin, etoposide, and melphalan as high-dose chemotherapy for high-risk neuroblastoma (HR-NBL1/SIOPEN): an international, randomised, multi-arm, open-label, phase 3 trial. Lancet Oncol. 2017;18: 500–514. 10.1016/S1470-2045(17)30070-0 28259608

[7] Pugh TJ, Morozova O, Attiyeh EF, Asgharzadeh S, Wei JS, Auclair D, et al The genetic landscape of high-risk neuroblastoma. Nat Genet. 2013;45: 279 284. 10.1038/ng.2529 23334666 PMC3682833

[8] Schleiermacher G, Janoueix-Lerosey I, Ribeiro A, Klijanienko J, Couturier J, Pierron G, et al Accumulation of Segmental Alterations Determines Progression in Neuroblastoma. J Clin Oncol. 2010;28: 3122 3130. 10.1200/JCO.2009.26.7955 20516441

[9] Brodeur GM, Seeger RC, Schwab M, Varmus HE, Bishop JM. Amplification of N-myc in untreated human neuroblastomas correlates with advanced disease stage. Science (New York, NY). 1984;224: 1121 1124. 10.1126/science.6719137 6719137

[10] Attiyeh EF, London WB, Mosse YP, Wang Q, Winter C, Khazi D, et al Chromosome 1p and 11q Deletions and Outcome in Neuroblastoma. New Engl J Medicine. 2005;353: 2243 2253. 10.1056/NEJMoa052399 16306521

[11] Spitz R, Hero B, Ernestus K, Berthold F. Deletions in chromosome arms 3p and 11q are new prognostic markers in localized and 4s neuroblastoma. Clinical cancer research: an official journal of the American Association for Cancer Research. 2003;9: 52 58. 12538451

[12] Schleiermacher G, Mosseri V, London WB, Maris JM, Brodeur GM, Attiyeh E, et al Segmental chromosomal alterations have prognostic impact in neuroblastoma: a report from the INRG project. Brit J Cancer. 2012;107: 1418 1422. 10.1038/bjc.2012.375 22976801 PMC3494425

[13] Janoueix-Lerosey I, Schleiermacher G, Michels E, Mosseri V, Ribeiro A, Lequin D, et al Overall Genomic Pattern Is a Predictor of Outcome in Neuroblastoma. J Clin Oncol. 2009;27: 1026 1033. 10.1200/JCO.2008.16.0630 19171713

[14] George RE, Sanda T, Hanna M, Fröhling S, II WL, Zhang J, et al Activating mutations in ALK provide a therapeutic target in neuroblastoma. Nature. 2008;455: 975 978. 10.1038/nature07397 18923525 PMC2587486

[15] Chen Y, Takita J, Choi YL, Kato M, Ohira M, Sanada M, et al Oncogenic mutations of ALK kinase in neuroblastoma. Nature. 2008;455: 971 974. 10.1038/nature07399 18923524

[16] Bresler SC, Weiser DA, Huwe PJ, Park JH, Krytska K, Ryles H, et al ALK Mutations Confer Differential Oncogenic Activation and Sensitivity to ALK Inhibition Therapy in Neuroblastoma. Cancer Cell. 2014;26: 682 694. 10.1016/j.ccell.2014.09.019 25517749 PMC4269829

[17] Janoueix-Lerosey I, Lequin D, Brugières L, Ribeiro A, Pontual L de, Combaret V, et al Somatic and germline activating mutations of the ALK kinase receptor in neuroblastoma. Nature. 2008;455: 967 970. 10.1038/nature07398 18923523

[18] Molenaar JJ, Koster J, Sluis P van, Zwijnenburg DA, Valentijn LJ, Ploeg I van der, et al Sequencing of neuroblastoma identifies chromothripsis and defects in neuritogenesis genes. Nature. 2012;483: 589 593. 10.1038/nature10910 22367537

[19] Cheung N-KV, Zhang J, Lu C, Parker M, Bahrami A, Tickoo SK, et al Association of Age at Diagnosis and Genetic Mutations in Patients With Neuroblastoma. Jama. 2012;307: 1062 1071. 10.1001/jama.2012.228 22416102 PMC3527076

[20] Valentijn LJ, Koster J, Zwijnenburg DA, Hasselt NE, Sluis P van, Volckmann R, et al TERT rearrangements are frequent in neuroblastoma and identify aggressive tumors. Nat Genet. 2015;47: 1411 1414. 10.1038/ng.3438 26523776

[21] Groningen T van Valentijn LJ, Koster J Zwijnenburg DA, Akogul N Hasselt NE, et al Neuroblastoma is composed of two super-enhancer-associated differentiation states. Nat Genet. 2017;49: 1261 1266. 10.1038/ng.3899 28650485

[22] Boeva V, Louis-Brennetot C, Peltier A, Durand S, Pierre-Eugène C, Raynal V, et al Heterogeneity of neuroblastoma cell identity defined by transcriptional circuitries. Nat Genet. 2017;49: 1408 1413. 10.1038/ng.3921 28740262

[23] Brodeur GM, Pritchard J, Berthold F, Carlsen NL, Castel V, Castelberry RP, et al Revisions of the international criteria for neuroblastoma diagnosis, staging, and response to treatment. J Clin Oncol. 1993;11: 1466 1477. 10.1200/JCO.1993.11.8.1466 8336186

[24] Sekiguchi M, Seki M, Kawai T, Yoshida K, Yoshida M, Isobe T, et al Integrated multiomics analysis of hepatoblastoma unravels its heterogeneity and provides novel druggable targets. Npj Precis Oncol. 2020;4: 20 10.1038/s41698-020-0125-y 32656360 PMC7341754

[25] Seki M, Kimura S, Isobe T, Yoshida K, Ueno H, Nakajima-Takagi Y, et al Recurrent SPI1 (PU.1) fusions in high-risk pediatric T cell acute lymphoblastic leukemia. Nat Genet. 2017;49: 1274 1281. 10.1038/ng.3900 28671687

[26] Kimura S, Hasegawa D, Yoshimoto Y, Seki M, Daida A, Sekiguchi M, et al Duplication of ALK F1245 missense mutation due to acquired uniparental disomy associated with aggressive progression in a patient with relapsed neuroblastoma. Oncol Lett. 2019;17: 3323 3329. 10.3892/ol.2019.9985 30867766 PMC6396392

[27] Shiraishi Y, Sato Y, Chiba K, Okuno Y, Yoshida K, Nagata Y, et al An empirical Bayesian framework for somatic mutation detection from cancer genome sequencing data. Nucleic Acids Res. 2013;41: e89 e89. 10.1093/nar/gkt126 23471004 PMC3627598

[28] Robinson JT, Thorvaldsdóttir H, Winckler W, Guttman M, Lander ES, Getz G, et al Integrative genomics viewer. Nat Biotechnol. 2011;29: 24 26. 10.1038/nbt.1754 21221095 PMC3346182

[29] Yoshizato T, Nannya Y, Atsuta Y, Shiozawa Y, Iijima-Yamashita Y, Yoshida K, et al Genetic abnormalities in myelodysplasia and secondary acute myeloid leukemia: impact on outcome of stem cell transplantation. Blood. 2017;129: 2347–2358. 10.1182/blood-2016-12-754796 28223278 PMC5409449

[30] Mylonas E, Yoshida K, Frick M, Hoyer K, Christen F, Kaeda J, et al Single-cell analysis based dissection of clonality in myelofibrosis. Nat Commun. 2020;11: 73 10.1038/s41467-019-13892-x 31911629 PMC6946829

[31] Harenza JL, Diamond MA, Adams RN, Song MM, Davidson HL, Hart LS, et al Transcriptomic profiling of 39 commonly-used neuroblastoma cell lines. Sci Data. 2017;4: 170033 10.1038/sdata.2017.33 28350380 PMC5369315

[32] Takagi M, Yoshida M, Nemoto Y, Tamaichi H, Tsuchida R, Seki M, et al Loss of DNA Damage Response in Neuroblastoma and Utility of a PARP Inhibitor. Jnci J National Cancer Inst. 2017;109 10.1093/jnci/djx062 29059438

[33] Ackermann S, Cartolano M, Hero B, Welte A, Kahlert Y, Roderwieser A, et al A mechanistic classification of clinical phenotypes in neuroblastoma. Science. 2018;362: 1165 1170. 10.1126/science.aat6768 30523111 PMC7875194

[34] Consortium TEP. An integrated encyclopedia of DNA elements in the human genome. Nature. 2012;489: 57 74. 10.1038/nature11247 22955616 PMC3439153

[35] Furlan A, Dyachuk V, Kastriti ME, Calvo-Enrique L, Abdo H, Hadjab S, et al Multipotent peripheral glial cells generate neuroendocrine cells of the adrenal medulla. Science. 2017;357: eaal3753 10.1126/science.aal3753 28684471 PMC6013038

[36] Riggelen J van, Yetil A, Felsher DW. MYC as a regulator of ribosome biogenesis and protein synthesis. Nat Rev Cancer. 2010;10: 301 309. 10.1038/nrc2819 20332779

[37] Lotharius J, Brundin P. Pathogenesis of parkinson’s disease: dopamine, vesicles and α-synuclein. Nat Rev Neurosci. 2002;3: 932 942. 10.1038/nrn983 12461550

[38] Muñoz P, Huenchuguala S, Paris I, Segura-Aguilar J. Dopamine Oxidation and Autophagy. Adv Neurol. 2012;2012: 1 13. 10.1155/2012/920953 22966478 PMC3433151

[39] Saint-André V, Federation AJ, Lin CY, Abraham BJ, Reddy J, Lee TI, et al Models of human core transcriptional regulatory circuitries. Genome Res. 2016;26: 385–396. 10.1101/gr.197590.115 26843070 PMC4772020

[40] Liu W, Le A, Hancock C, Lane A. N, Dang C. V, Fan T. W. M, et al Reprogramming of proline and glutamine metabolism contributes to the proliferative and metabolic responses regulated by oncogenic transcription factor c-MYC. Proc National Acad Sci. 2012;109: 8983 8988. 10.1073/pnas.1203244109 22615405 PMC3384197

[41] Loayza-Puch F, Rooijers K, Buil LCM, Zijlstra J, Vrielink JFO, Lopes R, et al Tumour-specific proline vulnerability uncovered by differential ribosome codon reading. Nature. 2016;530: 490 494. 10.1038/nature16982 26878238

[42] Volavka J, Bilder R, Nolan K. Catecholamines and Aggression: The Role of COMT and MAO Polymorphisms. Ann Ny Acad Sci. 2006;1036: 393 398. 10.1196/annals.1330.023 15817751

[43] Peaston RT, Weinkove C. Measurement of catecholamines and their metabolites. Ann Clin Biochem. 2004;41: 17 38. 10.1258/000456304322664663 14713382

[44] Maser T, Rich M, Hayes D, Zhao P, Nagulapally AB, Bond J, et al Tolcapone induces oxidative stress leading to apoptosis and inhibition of tumor growth in Neuroblastoma. Cancer Med-us. 2017;6: 1341 1352. 10.1002/cam4.1065 28429453 PMC5463066

[45] Temple W, Mendelsohn L, Kim GE, Nekritz E, Gustafson WC, Lin L, et al Vesicular monoamine transporter protein expression correlates with clinical features, tumor biology, and MIBG avidity in neuroblastoma: a report from the Children’s Oncology Group. Eur J Nucl Med Mol I. 2015;43: 474–481. 10.1007/s00259-015-3179-2 26338179 PMC4733400

