# Supplementary figures and images for: Association of high-risk neuroblastoma classification based on expression profiles with differentiation and metabolism
Source: PLoS One. 2021 Jan 19;16(1):e0245526. doi: 10.1371/journal.pone.0245526 (PMC7815088; doi:10.1371/journal.pone.0245526)

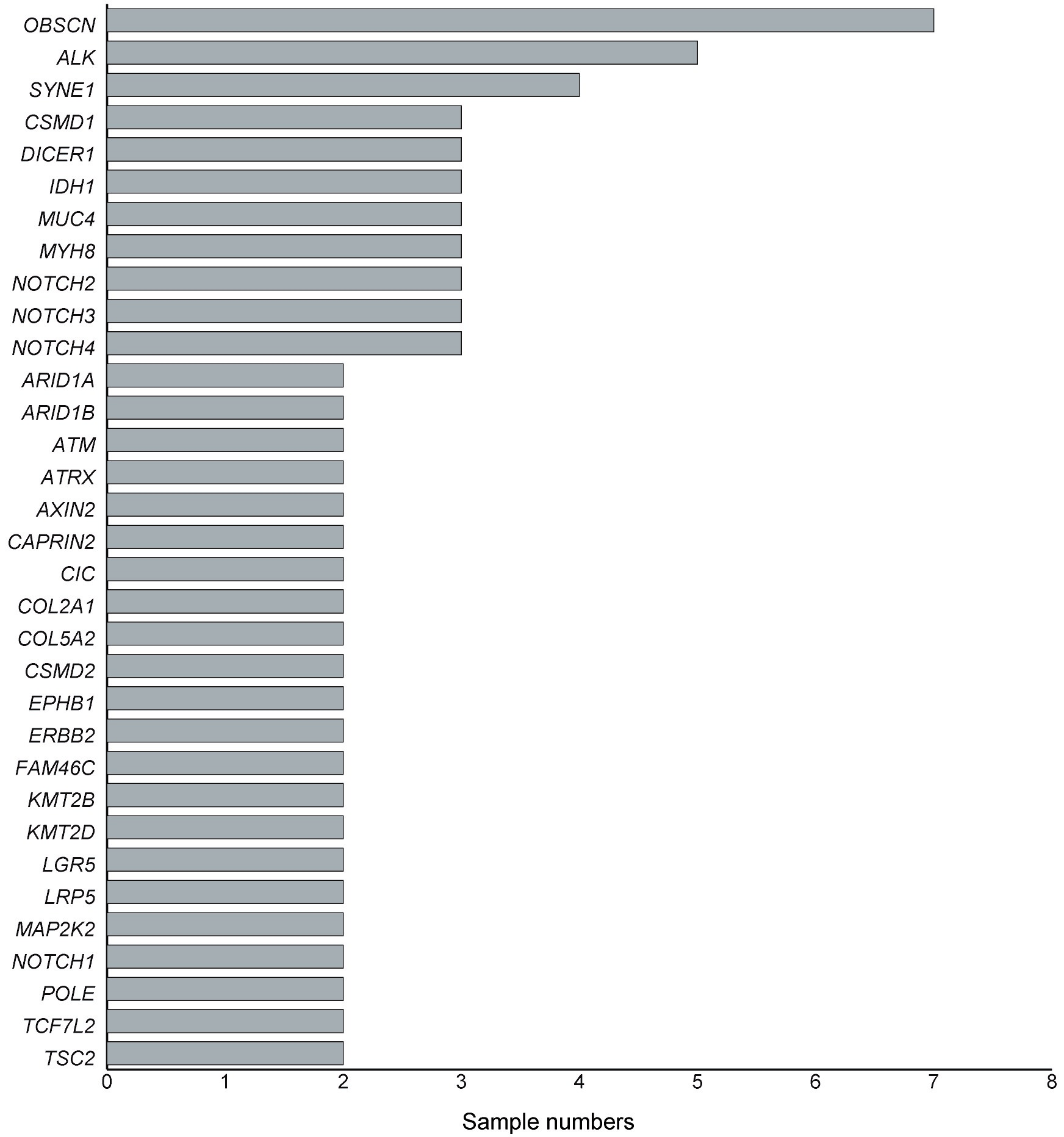

Supplement: S1 Fig — (TIF) [file pone.0245526.s001.tif]

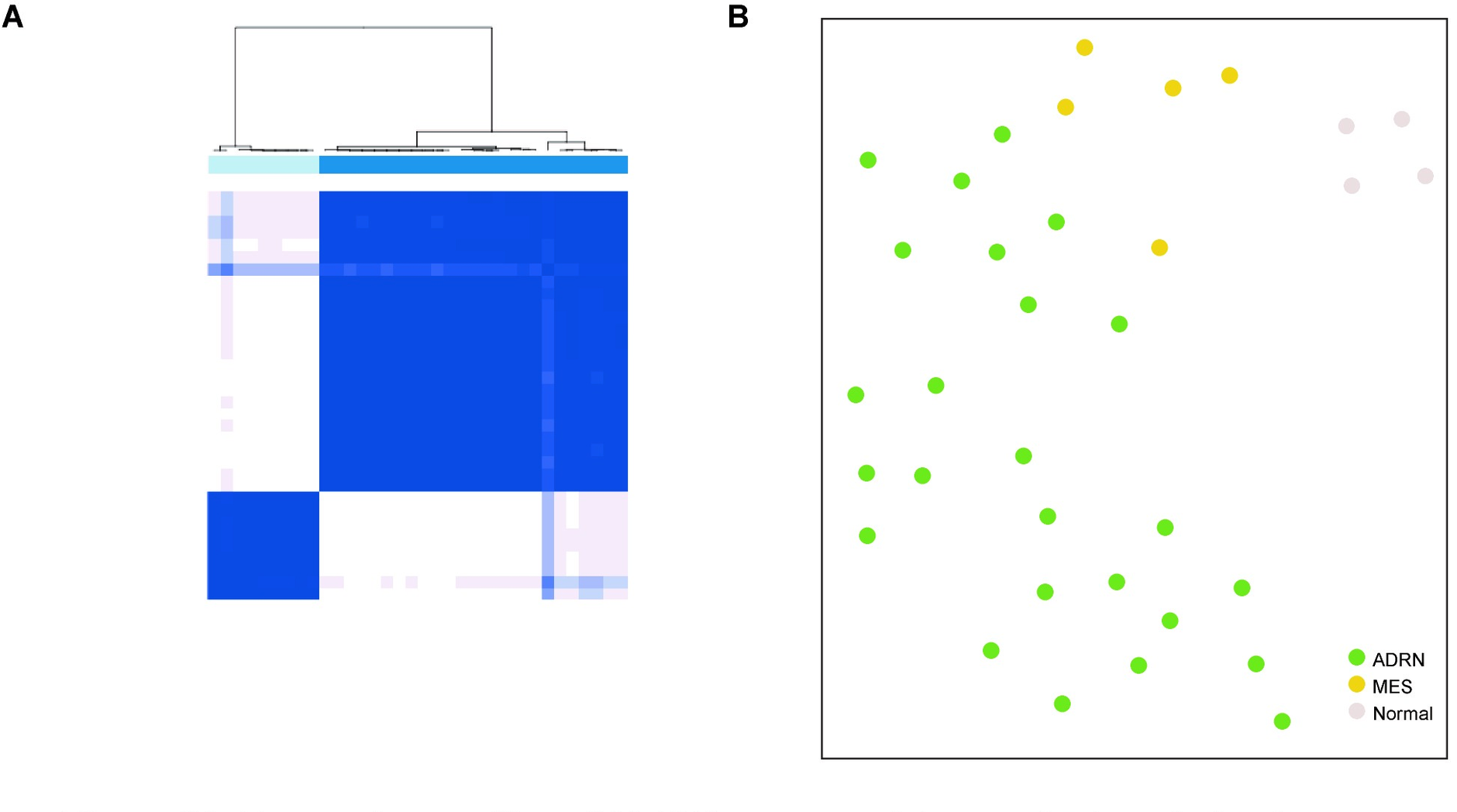

Supplement: S2 Fig — (TIF) [file pone.0245526.s002.tif]

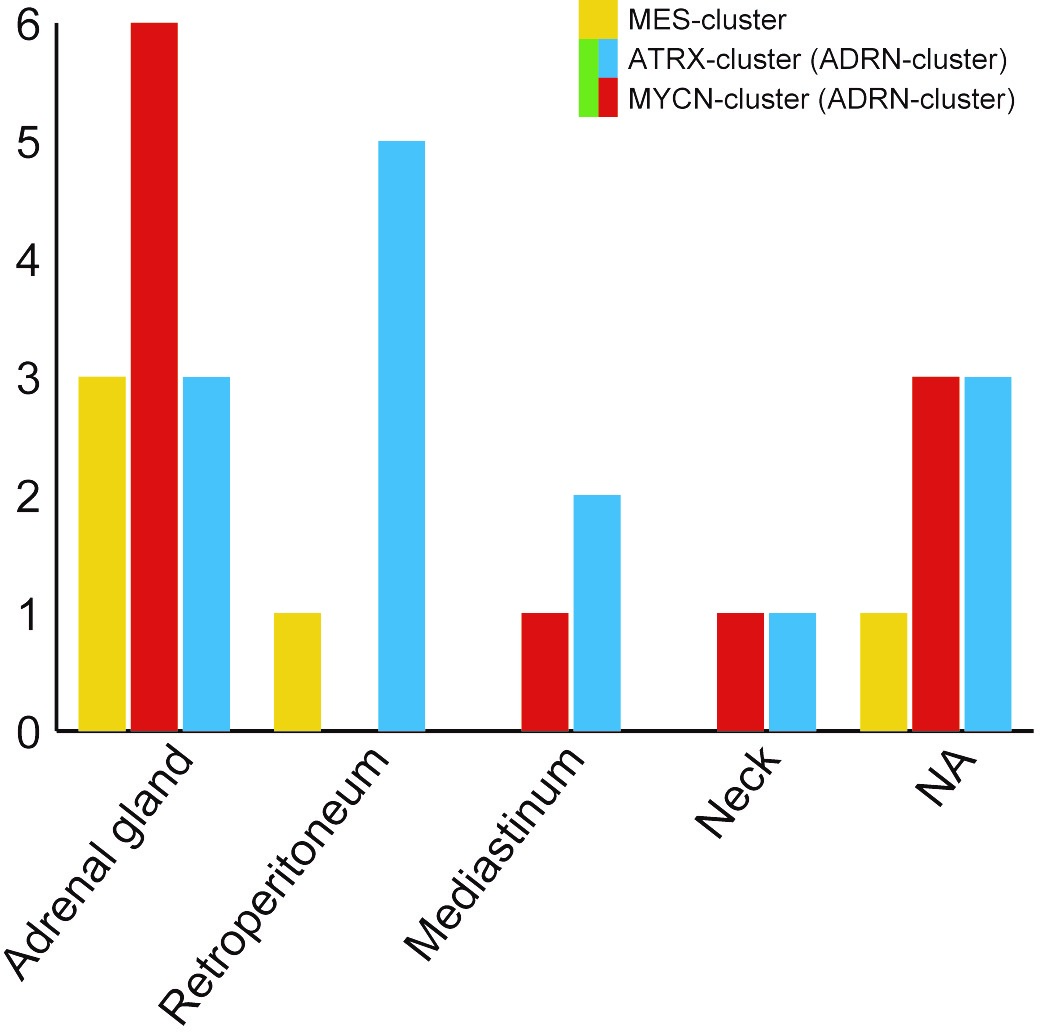

Supplement: S3 Fig — (TIF) [file pone.0245526.s003.tif]

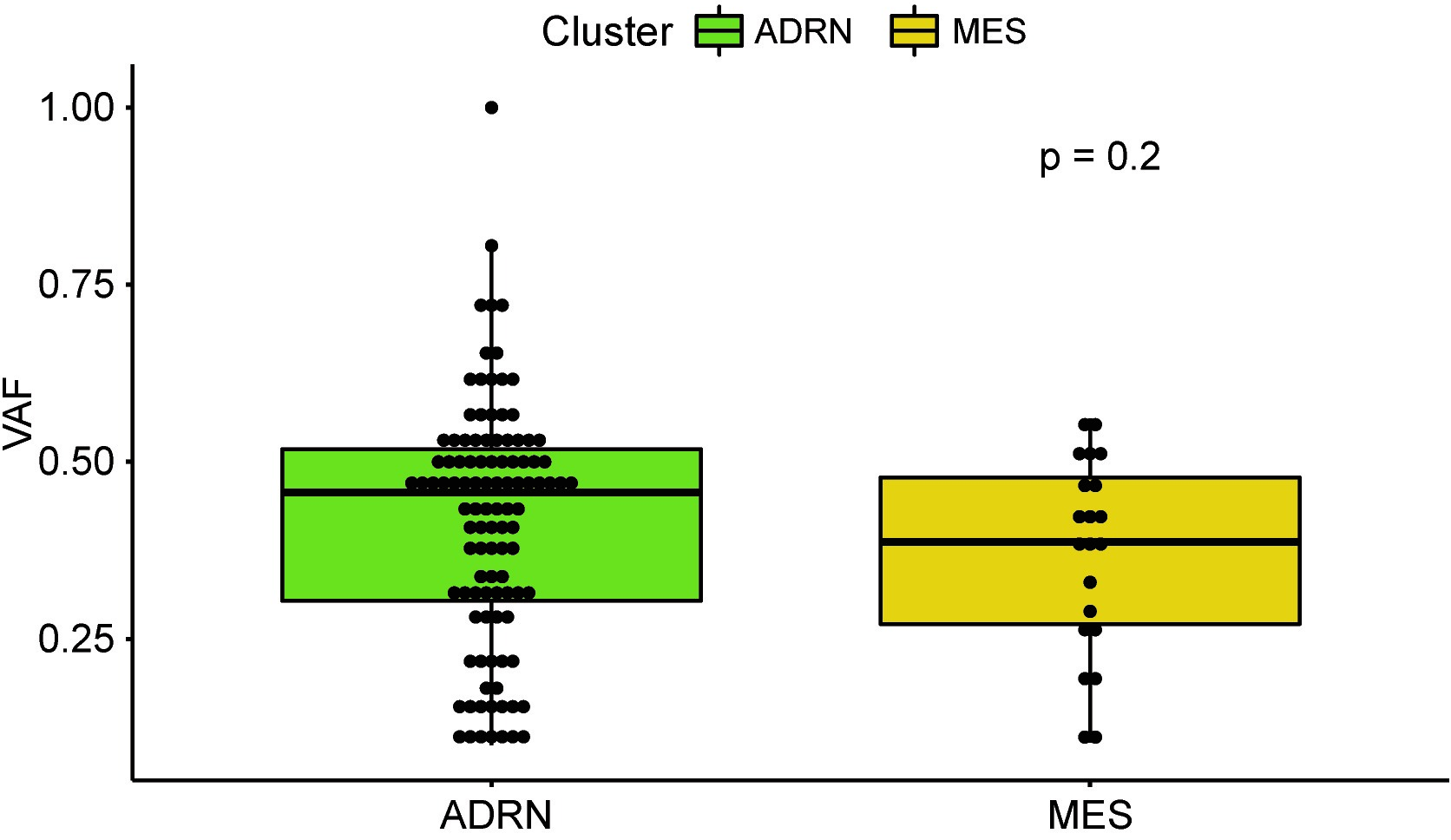

Supplement: S4 Fig — (TIF) [file pone.0245526.s004.tif]

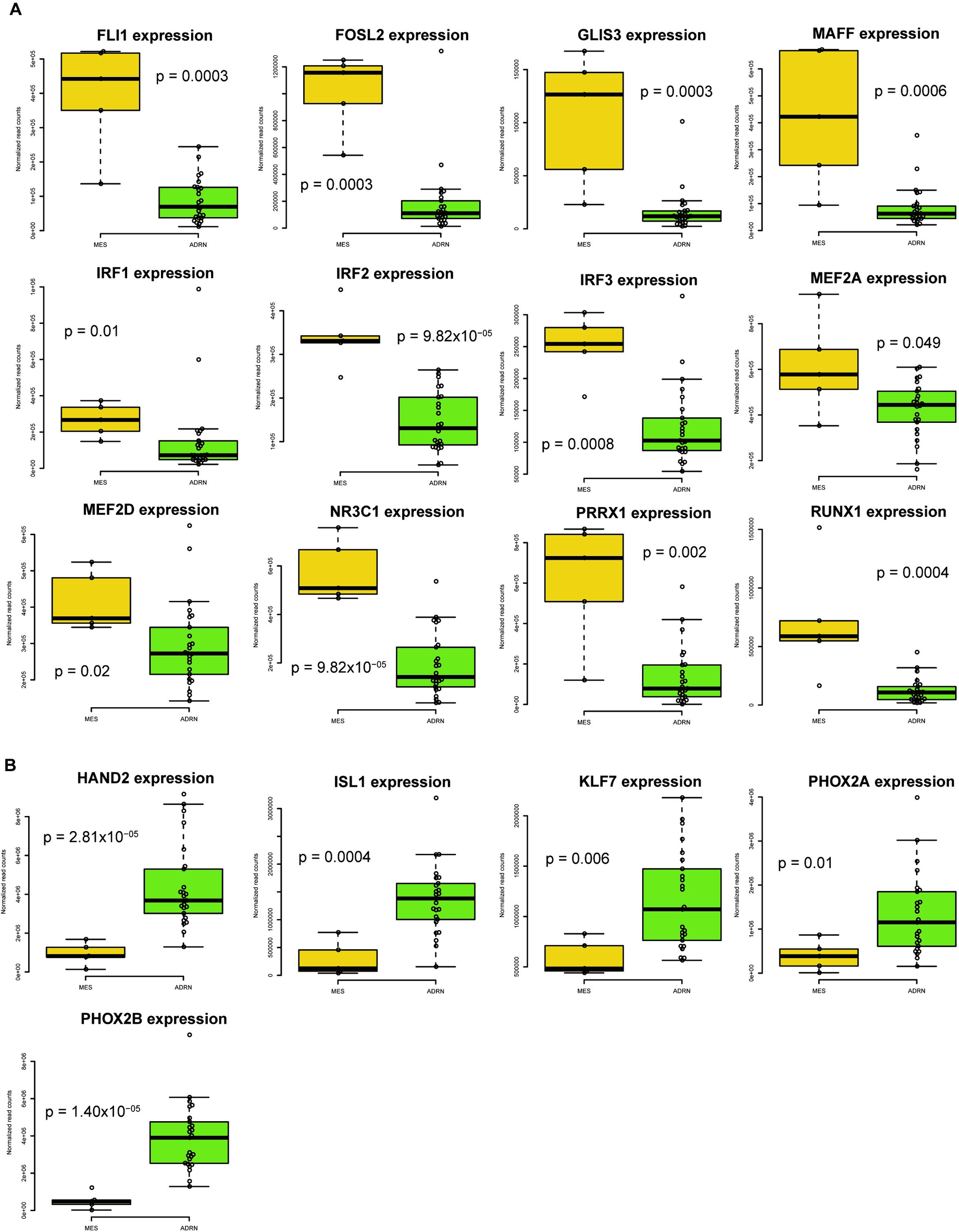

Supplement: S5 Fig — The expression of transcription factor genes participates in core regulatory circuitries in (A) MES-cluster and (B) ADRN-cluster. (TIF) [file pone.0245526.s005.tif]

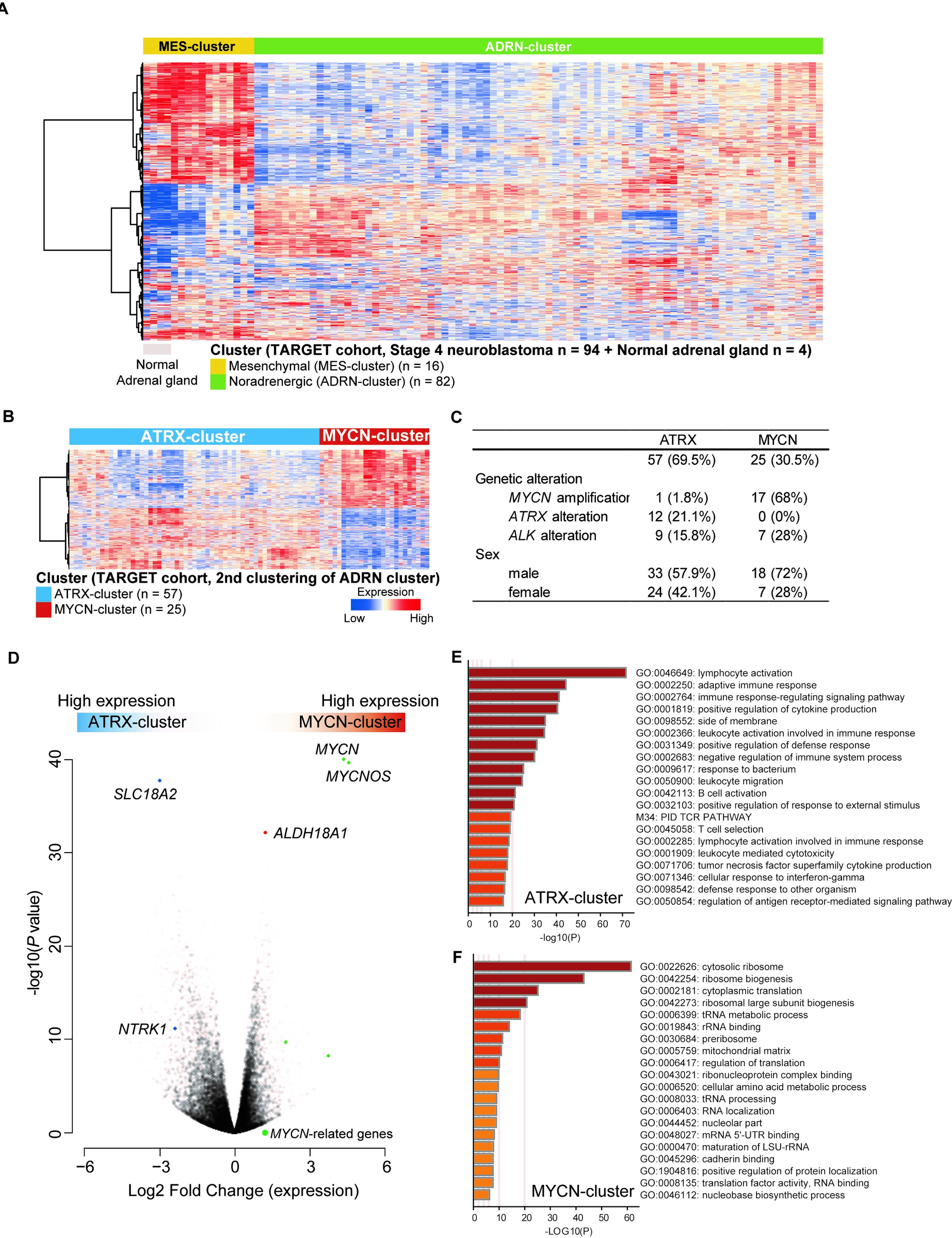

Supplement: S6 Fig — (TIF) [file pone.0245526.s006.tif]

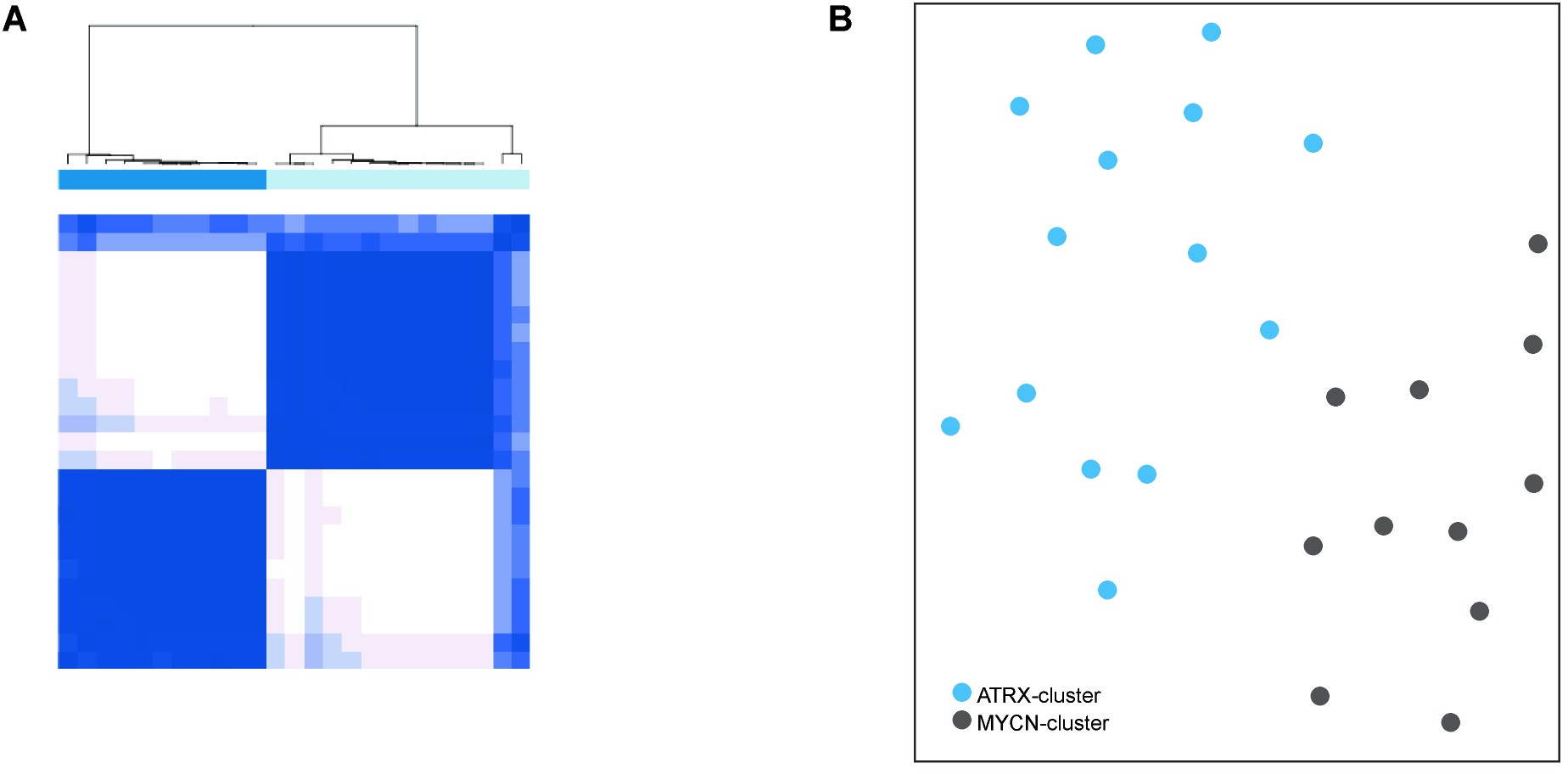

Supplement: S7 Fig — (TIF) [file pone.0245526.s007.tif]
